# Risk and protective factors of drug abuse among adolescents: a systematic review

**DOI:** 10.1186/s12889-021-11906-2

**Published:** 2021-11-13

**Authors:** Azmawati Mohammed Nawi, Rozmi Ismail, Fauziah Ibrahim, Mohd Rohaizat Hassan, Mohd Rizal Abdul Manaf, Noh Amit, Norhayati Ibrahim, Nurul Shafini Shafurdin

**Affiliations:** 1 Department of Community Health, Universiti Kebangsaan Malaysia, Cheras, 56000 Kuala Lumpur, Malaysia; 2 Centre for Research in Psychology and Human Well-Being (PSiTra), Faculty of Social Sciences and Humanities, Universiti Kebangsaan Malaysia, 43600 Bangi, Selangor Malaysia; 3 Clinical Psychology and Behavioural Health Program, Faculty of Health Sciences, Universiti Kebangsaan Malaysia, Kuala Lumpur, Malaysia

**Keywords:** Risk factor, Protective factor, Drug abuse, substance, adolescent

## Abstract

**Background:**

Drug abuse is detrimental, and excessive drug usage is a worldwide problem. Drug usage typically begins during adolescence. Factors for drug abuse include a variety of protective and risk factors. Hence, this systematic review aimed to determine the risk and protective factors of drug abuse among adolescents worldwide.

**Methods:**

Preferred Reporting Items for Systematic Reviews and Meta-Analyses (PRISMA) was adopted for the review which utilized three main journal databases, namely PubMed, EBSCOhost, and Web of Science. Tobacco addiction and alcohol abuse were excluded in this review. Retrieved citations were screened, and the data were extracted based on strict inclusion and exclusion criteria. Inclusion criteria include the article being full text, published from the year 2016 until 2020 and provided via open access resource or subscribed to by the institution. Quality assessment was done using Mixed Methods Appraisal Tools (MMAT) version 2018 to assess the methodological quality of the included studies. Given the heterogeneity of the included studies, a descriptive synthesis of the included studies was undertaken.

**Results:**

Out of 425 articles identified, 22 quantitative articles and one qualitative article were included in the final review. Both the risk and protective factors obtained were categorized into three main domains: individual, family, and community factors. The individual risk factors identified were traits of high impulsivity; rebelliousness; emotional regulation impairment, low religious, pain catastrophic, homework completeness, total screen time and alexithymia; the experience of maltreatment or a negative upbringing; having psychiatric disorders such as conduct problems and major depressive disorder; previous e-cigarette exposure; behavioral addiction; low-perceived risk; high-perceived drug accessibility; and high-attitude to use synthetic drugs. The familial risk factors were prenatal maternal smoking; poor maternal psychological control; low parental education; negligence; poor supervision; uncontrolled pocket money; and the presence of substance-using family members. One community risk factor reported was having peers who abuse drugs. The protective factors determined were individual traits of optimism; a high level of mindfulness; having social phobia; having strong beliefs against substance abuse; the desire to maintain one’s health; high paternal awareness of drug abuse; school connectedness; structured activity and having strong religious beliefs.

**Conclusion:**

The outcomes of this review suggest a complex interaction between a multitude of factors influencing adolescent drug abuse. Therefore, successful adolescent drug abuse prevention programs will require extensive work at all levels of domains.

## Introduction

Drug abuse is a global problem; 5.6% of the global population aged 15–64 years used drugs at least once during 2016 [1]. The usage of drugs among younger people has been shown to be higher than that among older people for most drugs. Drug abuse is also on the rise in many ASEAN (Association of Southeast Asian Nations) countries, especially among young males between 15 and 30 years of age. The increased burden due to drug abuse among adolescents and young adults was shown by the Global Burden of Disease (GBD) study in 2013 [2]. About 14% of the total health burden in young men is caused by alcohol and drug abuse. Younger people are also more likely to die from substance use disorders [3], and cannabis is the drug of choice among such users [4].

Adolescents are the group of people most prone to addiction [5]. The critical age of initiation of drug use begins during the adolescent period, and the maximum usage of drugs occurs among young people aged 18–25 years old [1]. During this period, adolescents have a strong inclination toward experimentation, curiosity, susceptibility to peer pressure, rebellion against authority, and poor self-worth, which makes such individuals vulnerable to drug abuse [2]. During adolescence, the basic development process generally involves changing relations between the individual and the multiple levels of the context within which the young person is accustomed. Variation in the substance and timing of these relations promotes diversity in adolescence and represents sources of risk or protective factors across this life period [6]. All these factors are crucial to helping young people develop their full potential and attain the best health in the transition to adulthood. Abusing drugs impairs the successful transition to adulthood by impairing the development of critical thinking and the learning of crucial cognitive skills [7]. Adolescents who abuse drugs are also reported to have higher rates of physical and mental illness and reduced overall health and well-being [8].

The absence of protective factors and the presence of risk factors predispose adolescents to drug abuse. Some of the risk factors are the presence of early mental and behavioral health problems, peer pressure, poorly equipped schools, poverty, poor parental supervision and relationships, a poor family structure, a lack of opportunities, isolation, gender, and accessibility to drugs [9]. The protective factors include high self-esteem, religiosity, grit, peer factors, self-control, parental monitoring, academic competence, anti-drug use policies, and strong neighborhood attachment [10–15].

The majority of previous systematic reviews done worldwide on drug usage focused on the mental, psychological, or social consequences of substance abuse [16–18], while some focused only on risk and protective factors for the non-medical use of prescription drugs among youths [19]. A few studies focused only on the risk factors of single drug usage among adolescents [20]. Therefore, the development of the current systematic review is based on the main research question: What is the current risk and protective factors among adolescent on the involvement with drug abuse? To the best of our knowledge, there is limited evidence from systematic reviews that explores the risk and protective factors among the adolescent population involved in drug abuse. Especially among developing countries, such as those in South East Asia, such research on the risk and protective factors for drug abuse is scarce. Furthermore, this review will shed light on the recent trends of risk and protective factors and provide insight into the main focus factors for prevention and control activities program. Additionally, this review will provide information on how these risk and protective factors change throughout various developmental stages. Therefore, the objective of this systematic review was to determine the risk and protective factors of drug abuse among adolescents worldwide. This paper thus fills in the gaps of previous studies and adds to the existing body of knowledge. In addition, this review may benefit certain parties in developing countries like Malaysia, where the national response to drugs is developing in terms of harm reduction, prison sentences, drug treatments, law enforcement responses, and civil society participation.

## Methods

This systematic review was conducted using three databases, PubMed, EBSCOhost, and Web of Science, considering the easy access and wide coverage of reliable journals, focusing on the risk and protective factors of drug abuse among adolescents from 2016 until December 2020. The search was limited to the last 5 years to focus only on the most recent findings related to risk and protective factors. The search strategy employed was performed in accordance with the Preferred Reporting Items for a Systematic Review and Meta-analysis (PRISMA) checklist.

A preliminary search was conducted to identify appropriate keywords and determine whether this review was feasible. Subsequently, the related keywords were searched using online thesauruses, online dictionaries, and online encyclopedias. These keywords were verified and validated by an academic professor at the National University of Malaysia. The keywords used as shown in Table 1.

**Table 1:** The search strings

Database Search string	
PubMed	adolescent OR teenager OR teen OR youth OR school-going children OR youngster OR pediatric* AND abuse OR addiction OR dependence OR habituation OR overdose OR misuse OR overuse OR use AND drug OR narcotic OR opioid OR psychoactive substance OR amphetamine OR cannabis OR ecstasy OR heroin OR cocaine OR hallucinogen* OR depressant OR stimulant OR marijuana OR illicit drug OR tranquilizers OR sedatives OR LSD OR Fentanyl OR illegal drug OR street drug OR club drug OR recreational drug OR substances AND risk factor OR protective factor OR predictive factor OR determinant OR cause
EBSCOhost	TX (“adolescent” OR “teenager” OR “teen’ OR youth” OR “school-going children” OR “youngster” OR pediatric) AND TX (“abuse” OR “addiction” OR “dependence” OR “habituation” OR “overdose” OR “misuse” OR “overuse” OR “use”) AND TX (“drug” OR “narcotic” OR “opioid” OR “psychoactive substance” OR “amphetamine” OR “cannabis” OR “ecstasy” OR “heroin” OR “cocaine” OR “hallucinogens” OR “depressant” OR “stimulant” OR “marijuana” OR “illicit drug” OR “tranquilizers” OR “sedatives” OR “LSD” OR “Fentanyl” OR “illegal drug” OR “street drug” OR “recreational drug” OR “substances”) AND TX (“risk factor” OR “protective factor” OR “predictive factor” OR “determinant” OR “cause”)
WoS	TS = (((“adolescent” OR “teenager” OR “teen’ OR youth” OR “school-going children” OR “youngster” OR pediatric*) AND (“abuse” OR “ad-diction” OR “dependence” OR “habituation” OR “overdose” OR “misuse” OR “overuse” OR “use*”) AND (“drug” OR “narcotic” OR “opioid” OR “psychoactive substance” OR “amphetamine” OR “cannabis” OR “ecstasy” OR “heroin” OR “cocaine” OR “hallucinogens” OR “depressant” OR “stimulant” OR “marijuana” OR “illicit drug” OR “tranquilizers” OR “sedatives” OR “LSD” OR “Fentanyl” OR “illegal drug” OR “street drug” OR “recreational drug” OR “sub-stances”) AND (“risk factor” OR “protective factor” OR “predictive factor” OR “determinant” OR “cause”)

### Selection criteria

**Fig. 1:**
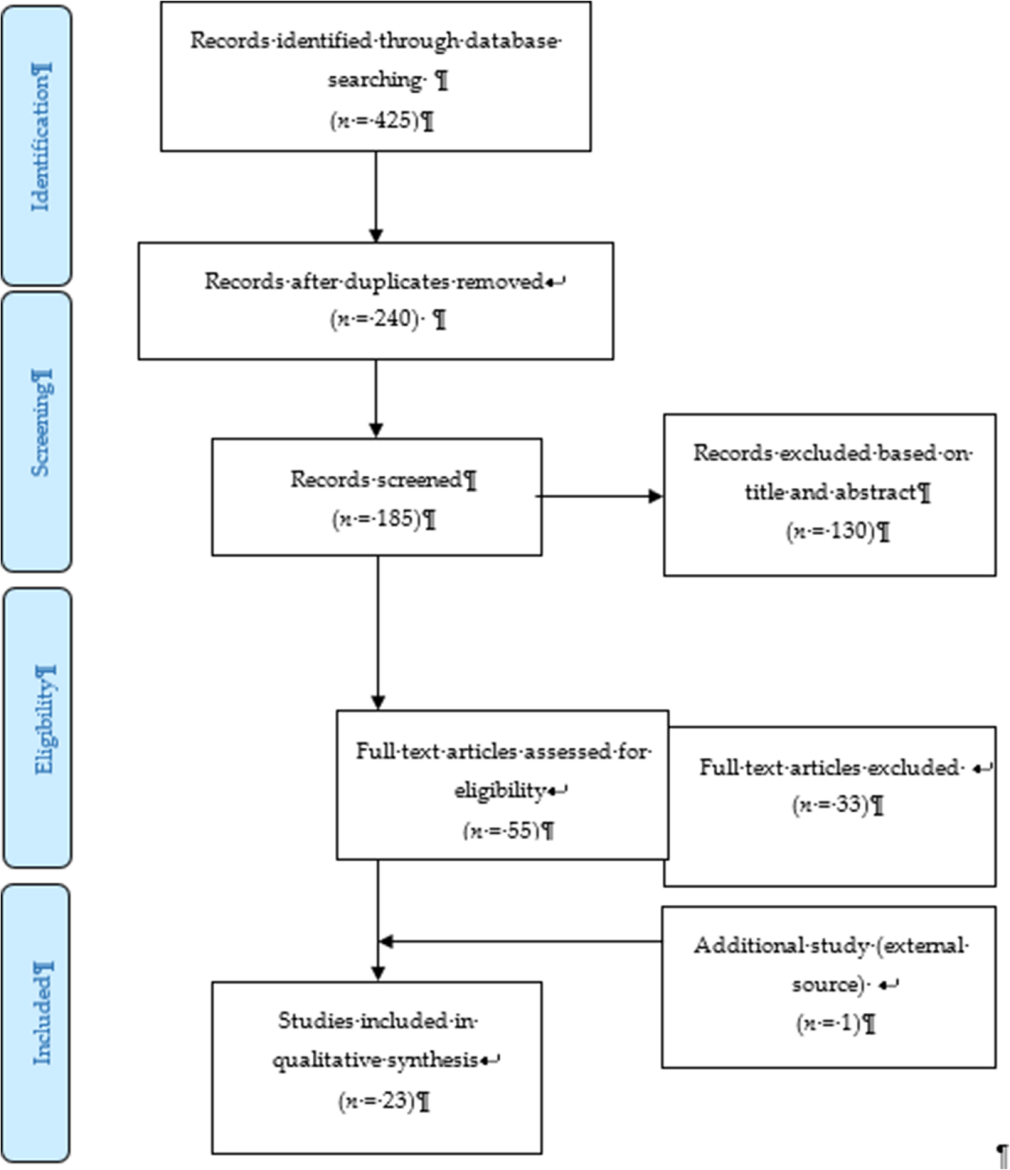
PRISMA flow diagram showing the selection of studies on risk and protective factors for drug abuse among adolescents.2.2. Operational Definition

The systematic review process for searching the articles was carried out via the steps shown in Fig. 1. Firstly, screening was done to remove duplicate articles from the selected search engines. A total of 240 articles were removed in this stage. Titles and abstracts were screened based on the relevancy of the titles to the inclusion and exclusion criteria and the objectives. The inclusion criteria were full text original articles, open access articles or articles subscribed to by the institution, observation and intervention study design and English language articles. The exclusion criteria in this search were (a) case study articles, (b) systematic and narrative review paper articles, (c) non-adolescent-based analyses, (d) non-English articles, and (e) articles focusing on smoking (nicotine) and alcohol-related issues only. A total of 130 articles were excluded after title and abstract screening, leaving 55 articles to be assessed for eligibility. The full text of each article was obtained, and each full article was checked thoroughly to determine if it would fulfil the inclusion criteria and objectives of this study. Each of the authors compared their list of potentially relevant articles and discussed their selections until a final agreement was obtained. A total of 22 articles were accepted to be included in this review. Most of the excluded articles were excluded because the population was not of the target age range—i.e., featuring subjects with an age > 18 years, a cohort born in 1965–1975, or undergraduate college students; the subject matter was not related to the study objective—i.e., assessing the effects on premature mortality, violent behavior, psychiatric illness, individual traits, and personality; type of article such as narrative review and neuropsychiatry review; and because of our inability to obtain the full article—e.g., forthcoming work in 2021. One qualitative article was added to explain the domain related to risk and the protective factors among the adolescents.

Drug-related substances in this context refer to narcotics, opioids, psychoactive substances, amphetamines, cannabis, ecstasy, heroin, cocaine, hallucinogens, depressants, and stimulants. Drugs of abuse can be either off-label drugs or drugs that are medically prescribed. The two most commonly abused substances not included in this review are nicotine (tobacco) and alcohol. Accordingly, e-cigarettes and nicotine vape were also not included. Further, “adolescence” in this study refers to members of the population aged between 10 to 18 years [21].

### Data extraction tool

All researchers independently extracted information for each article into an Excel spreadsheet. The data were then customized based on their (a) number; (b) year; (c) author and country; (d) titles; (e) study design; (f) type of substance abuse; (g) results—risks and protective factors; and (h) conclusions. A second reviewer crossed-checked the articles assigned to them and provided comments in the table.

### Quality assessment tool

By using the Mixed Method Assessment Tool (MMAT version 2018), all articles were critically appraised for their quality by two independent reviewers. This tool has been shown to be useful in systematic reviews encompassing different study designs [22]. Articles were only selected if both reviewers agreed upon the articles’ quality. Any disagreement between the assigned reviewers was managed by employing a third independent reviewer. All included studies received a rating of “yes” for the questions in the respective domains of the MMAT checklists. Therefore, none of the articles were removed from this review due to poor quality. The Cohen’s kappa (agreement) between the two reviewers was 0.77, indicating moderate agreement [23].

## Results

The initial search found 425 studies for review, but after removing duplicates and applying the criteria listed above, we narrowed the pool to 22 articles, all of which are quantitative in their study design. The studies include three prospective cohort studies [24–26], one community trial [27], one case-control study [28], and nine cross-sectional studies [29–45]. After careful discussion, all reviewer panels agreed to add one qualitative study [46] to help provide reasoning for the quantitative results. The selected qualitative paper was chosen because it discussed almost all domains on the risk and protective factors found in this review.

A summary of all 23 articles is listed in Table 2. A majority of the studies (13 articles) were from the United States of America (USA) [25–27, 29–31, 34, 36–45], three studies were from the Asia region [32, 33, 38], four studies were from Europe [24, 28, 40, 44], and one study was from Latin America [35], Africa [43] and Mediterranean [45]. The number of sample participants varied widely between the studies, ranging from 70 samples (minimum) to 700,178 samples (maximum), while the qualitative paper utilized a total of 100 interviewees. There were a wide range of drugs assessed in the quantitative articles, with marijuana being mentioned in 11 studies, cannabis in five studies, and opioid (six studies). There was also large heterogeneity in terms of the study design, type of drug abused, measurements of outcomes, and analysis techniques used. Therefore, the data were presented descriptively.

**Table 2:** Study characteristic and main findings

No	Year	Authors/ Country	Study objectives	Study design	Types of substance abuse	Result / findings Risk factors /Protective factors	Conclusion
1	2020	Dash et al. (USA)	To capture a time-sensitive report of the intersection of prescription opioid receipt and contextual risks for opioid misuse related to pain experience, mental health symptoms, and substance use at the adolescent and parental levels.	Cross-sectional	Opiod	Risk Factors 1) Pain catastrophe 2) Mother history of chronic pain (parents reported keeping opioids at home) and parent anxiety	Opioids at home as a risk factors for adolescent misuse
2	2020	Osborne et al. (USA)	To examine peer influence and parental guidance, in addition to peer and parental sources of alcohol, on patterns of prescription opioid use	Cross-sectional	Opiod	Risk factors 1) Close friend who used other substances 2) Alcoholic parents Protective Factors 1) Increased number of close friends	Increased number of close friends was a protective factor against prescription opioid
3	2020	Zuckermann et al.(Canada)	To investigate demographic and behavioral risk factors for non-medical use of prescription opioids.	Cross-sectional study	Opiod: oxycodone, fentanyl, other prescription pain relievers	Risk factors 1) lack of homework completion Protective Factors 1) School connectedness	School connectedness may lower the risk of non-medical use of prescription opioids, indicating that a school-based focus is justified.
4	2020	Spillane et al. (USA)	To examines the role of perceived availability and engagement in structured and unstructured activities on adolescent alcohol and marijuana use controlling for substance availability	Cross sectional	Marijuana	Risk Factors 1) Availability of unstructured activities	Perceived availability of and engagement in unstructured activities may present a risk, while perceived availability of and engagement in structured activities may serve as a protective factor for youth substance use
5	2020	Afifi et al.(Beirut)	To explore the association between bullying victimization and substance use in adolescents with low and high levels of religiosity.	Cross-sectional	Substance use	Risk Factors 1) Lower religiosity levels who had been bullied	Religiosity may be a potential moderator of the association between being bullied and substance use
6	2019	Marin S et al. (Iran)	To examine the relationship between optimistic explanatory style and cigarette smoking, hookah smoking, and illicit drug use among high school students in Sonqor county, Iran	Cross-sectional	Opium Cannabis Ecstasy Methamphetamine	Protective Factors 1) Optimism trait of an individual measured using Children Attributional Style Questionnaire (CASQ). 2) Higher scores of optimism protected students from using illicit drugs (Model 3: OR = 0.90, 95% CI: 0.85–0.95, *P* < 0.001). 3) Negative-stability and negative-globality domains of optimism were significantly higher among advanced-stage smokers and illicit drug users.	Optimism was found to be a protective factor against substance abuse.
7	2019	Schleimer et al. (Latin America: Chile, Uruguay, and Argentina)	1) To estimate associations between perceived availability and perceived risk of marijuana use and past-month marijuana use 2) To describe how these associations changed over time	Cross-sectional	Marijuana	Risk Factors 1) No/ Low perceived risk increase the odds of past-month marijuana use by 8.22 times compared to those who perceived moderate/great risk. 2) High perceived availability of drug: consistently associated with higher odds of past-month marijuana use. Protective Factors 1) Moderate/ High perceived risk of substance use. 2) Low perceived availability	Perceived risk and availability of marijuana are significant risk factors for adolescent marijuana use in the Southern Cone.
8	2019	Guttmannova et al. (USA)	To examine a set of marijuana-specific risk factors from multiple domains of development for marijuana use over the course of adolescence	Community Randomized-Controlled Trial	Marijuana	Risk Factors 1) Perception of lax community enforcement of marijuana laws regarding adolescent use 2) Low perception of harm 3) Rebelliousness traits 4) Parents with low education	A greater frequency of marijuana use was predicted among the identified risk factors.
9	2019	Doggett et al. (Canada)	To examine the association between various types of screen time sedentary behavior (STSBs) and cannabis use	Cross-sectional	Cannabis	Risk Factors 1) Total screen time sedentary behavior (internet use, messaging, playing video games, watching TV	STSB is a risk factor for the tendency for individuals to use substances as a coping mechanism.
10	2017	Wilson et al. (USA)	To examine associations among levels of trait mindfulness and opioid use behaviors.	Cross sectional	Opioid	- Study using a convenience sample of 112 youth (ages 14–24) was recruited during an episode of inpatient detoxification and residential treatment for opioid use disorders. - Youth had difficulties in emotion regulation (m = 104.2; SD = 2.41) and low mindfulness (m = 19.1; SD = 0.59). Risk Factors 1) Difficulty in regulating emotions Protective Factors 1) High level of mindfulness	Majority of youth presenting with opioid use disorders have impairments in emotion regulation and deficits in trait mindfulness.
11	2017	Li et al. (Macau)	To identify culturally relevant predictors of synthetic drug use among adolescents in Macao.	Cross sectional	Ketamine Ecstasy/MDMA Methamphetamine Tranquilizers Hybrid synthetic drugs	- The rates of synthetic use among male adolescents were higher than those among female adolescents for lifetime use (1.79% vs. 1.04%), past-year use (1.29% vs. 0.70%), and past-month use (1.03% vs. 0.44%). - Synthetic drug use was the most prevalent among fifth and sixth graders at the elementary school level. Risk Factors 1) Peer usage 2) Recreational use of time 3) Attitudes towards synthetic drugs 4) Availability of synthetic drugs	The investigated risk factors contribute to adolescent drug abuse.
12	2017	Luk et al. (USA)	To examine both direct and indirect effects of multiple parenting dimensions on substance use behaviors across Asian-Pacific Islander (API) and European American youth.	Prospective Cohort	Marijuana	- Mother’s knowledge predicted fewer externalizing problems in Grade 8, which in turn predicted fewer substance use problems in Grades 9 and 12. - Father’s warmth predicted better academic achievement in Grade 8, which in turn predicted fewer substance use problems in Grades 9 and 12, as well as alcohol and marijuana dependence in Grade 12. Risk Factors 1) Mother’s psychological control Protective Factors 1) Father’s knowledge	Promoting father’s knowledge of adolescents’ whereabouts can reduce substance use risks among both European and API Americans.
13	2017	De Pedro et al. (USA)	This study aims to fill this gap in the literature and inform programs aimed at reducing substance use among LGB youth	Cross-sectional	Marijuana, inhalants, prescription pain medication, and other illegal drugs	Protective Factors 1) school connectedness and school adult support	The results indicate a need for substance use prevention programs that integrate school connectedness and adult support in school
14	2017	Dorard et al. (France)	To investigate alexithymia in young outpatient cannabis misusers to determine whether the levels of alexithymia and the state and traits of anxiety and depression predict cannabis misuse by adolescents	Case control	Cannabis	- Study done on 120 young patients with cannabis dependence or abuse (DSM-IV-TR criteria evaluated with the MINI) and seeking treatment in an addiction unit + another 110 healthy control subjects. - Used self-reports for measuring alexithymia (TAS-20;BVAQ-B), depression (BDI-13), and states and traits of anxiety (STAI). - 35.3% of cannabis users were alexithymia Risk Factors 1) Difficulty in identifying feelings Protective Factors 1) Difficulty in describing feelings	Lower rate of alexithymics than in previous reports among substance abusers but higher than those reported in the control
15	2017	Kobulsky (USA)	To examine the relations between child physical and sexual abuse and early substance use among youths investigated by child protective services	Cohort	Marijuana Inhalants Hard drugs NMPD	- Significant indirect effects of physical abuse severity on early substance use were found through externalizing behavior problems in girls, with a significantly stronger relation found only between externalizing problems and early substance use in girls. Risk Factors 1) Girls: Physical abuse severity, externalizing problems	Significant gender differences in the effect of early substance from physical abuse.
16	2017	Chuang et al. (USA)	To examine the potential relationship between two self-reported risk factors (impulsivity and the presence of one or more behavioral addictions) and tobacco, alcohol, and marijuana use—or susceptibility to use these drugs in the future among nonusers—in an adolescent population	Cross-sectional	Marijuana	- Adolescents who had either impulsivity alone or at least two behavioral addictions alone were more likely to have used tobacco, alcohol, or marijuana compared to individuals who had neither risk factor (OR = 2.50–4.13), and- Individuals who endorsed both impulsivity and three or more behavioral addictions were the most likely to have used these drugs (OR = 9.40–10.13) Risk Factors 1) High impulsivity combined with more than 3 behavioral addictions.	High impulsivity was related to behavioral addictions in adolescents, and a combination of these two factors increased risk for drug use
17	2016	Khoddam, et al. (USA)	To study whether the relationship of conduct problems and several internalizing disorders with future substance use is redundant, incremental, or interactive in adolescents.	Cross-sectional	Marijuana	Risk Factors 1) Conduct Problems (CPs) 2) Major depressive disorder Protective Factors 1) Social phobia	CPs are a risk factor for substance use, as well as the nuanced interplay of internalizing-externalizing problems in the developmental psychopathology of adolescent drug use vulnerability.
18	2016	Gabrielli et al. (USA)	To identify the relations between maltreatment and SU behavior in a population known for a significant risk of SU behaviour—youth in foster care.	Cross-sectional	Alcohol Marijuana Cocaine Stimulants LSD Tranquilizers Opiates PCP Sniffed gases/fumes Prescribed drugs	- 31% of participants reported past-year substance abuse. - Age of substance abuse onset was 11.08 years (Sd = 2.21 years) - Structural model with maltreatment predicting substance abuse severity demonstrated strong model fit with a significant path between maltreatment and substance abuse. Risk Factors 1) Maltreatment during stay in foster care.	Findings revealed a robust relationship between maltreatment, indicated by the severity and chronicity of experiences across types of maltreatment and substance use behavior severity.
19	2016	Traube et al. (USA)	1) To untangle two aspects of time in the growth process of polysubstance use: age or development and the length of time in the Child Welfare System (CWS). 2) To determine residential status as either a risk or protective factor	Cross-sectional	Alcohol Marijuana	- Analysis using longitudinal data from the National Survey of Child and Adolescent Well-Being (*n* = 1178). - Time- invariant characteristics of ethnicity and gender were not related to polysubstance use. - Increased proportions of the sample reporting the use of alcohol and marijuana (from 16 to 26% and from 9 to 18%, respectively). Risk Factors 1) Duration of stay in Child Welfare System (CWS)	Findings indicated that children who enter child welfare when they are older than age 15 are at increased risk of substance use, although those who enter the CWS at a young age may be at greater risk over time.
20	2016	Cecil et al. (UK)	1) To determine DNAm patterns at birth that are associated with adolescent substance use? 2) To identify DNAm markers that are associated with genetic and environmental influences	Cohort	Cannabis	- The sample comprised 244 youth (51% female) from the Avon Longitudinal Study of Parents and Children (ALSPAC). - At birth, epigenetic variation across a tightly interconnected genetic network (*n* = 65 loci; qo0.05) was associated with greater levels of substance use during adolescence, as well as an earlier age of onset among users. - Several of the identified loci were associated with known methylation quantitative trait loci. - Collectively, these 65 loci were also found to partially mediate the effect of prenatal maternal tobacco smoking on adolescent substance use. Risk Factors 1) Prenatal tobacco smoking	Tobacco exposure during pregnancy may increase the risk of future substance use.
21	2016	Ogunsola et al. (Nigeria)	To compare the prevalence of substance use among in-school adolescents in urban and rural areas of Osun State, Nigeria, and identified risk and protective factors.	Cross-sectional	Substances use	Risk Factors 1) Private school attendance 2) having friends who use substances 3) mother having had tertiary education Protective Factors 1) Parental disapproval of substance use	The risk and protective factors for adolescent substance use somewhat differ for rural and urban areas
22	2015	Miech et al. (USA)	To determine whether e-cigarette use is part of a pattern towards extensive substance use.	Cross-sectional	Marijuana Prescription drugs	- The distribution of e-cigarette use is consistent with the distribution of most other substances. - Youth who use e-cigarettes are, on average, highly likely to use other substances, as well. Risk Factors 1) E-cigarette smokers	Exposure to e-cigarettes within the past 30-days, increases the prevalence of marijuana use and prescription drug use among adolescents.
23	2018	El Kazdouh et al. (Morocco)	To explore and understand factors that protect or influence substance use in adolescents.	Focus Group Discussion (FGD) analysis via Thematic Analysis	Any illicit drug	Risk Factors 1) Perceived benefits of drug abuse 2) Perceived availability of drugs (cheaper price) 3) Lack of parental supervision 4) Peer pressure from those who do drugs Protective Factors 1) Strong belief in maintaining good health 2) Good family support in giving advice 3) Strong religious beliefs	There are many interplay factors that contribute to the risk of developing drug abuse problems and protecting adolescents from drug abuse. Key prevention activities need to be targeted at each level to ensure healthy behaviors among adolescents.

**Fig. 2:**
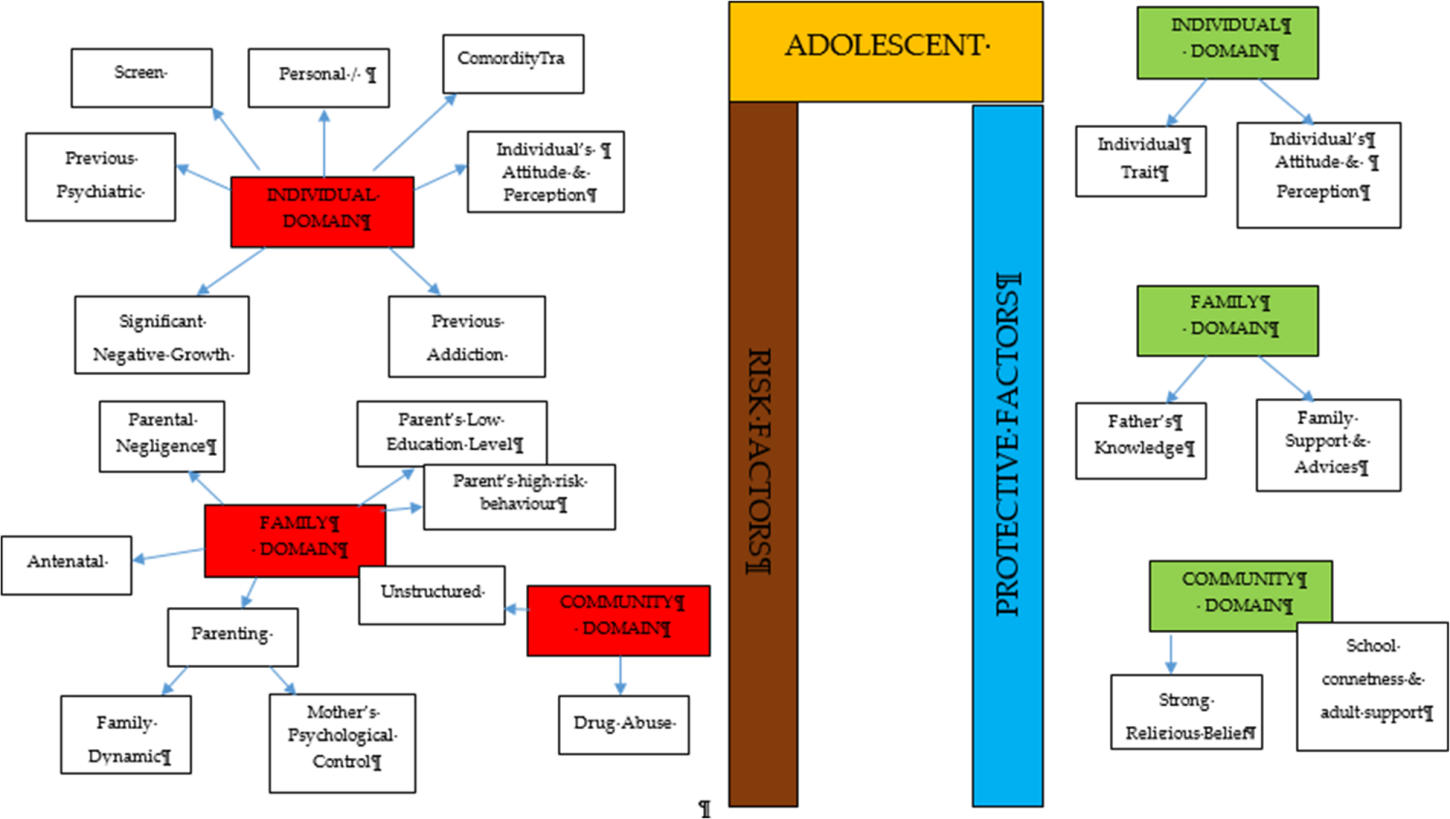
Conceptual framework of risk and protective factors related to adolescent drug abuse

After thorough discussion and evaluation, all the findings (both risk and protective factors) from the review were categorized into three main domains: individual factors, family factors, and community factors. The conceptual framework is summarized in Fig. 2.

### DOMAIN: individual factor

#### Risk factors

Almost all the articles highlighted significant findings of individual risk factors for adolescent drug abuse. Therefore, our findings for this domain were further broken down into five more sub-domains consisting of personal/individual traits, significant negative growth exposure, personal psychiatric diagnosis, previous substance history, comorbidity and an individual’s attitude and perception.

#### Personal/individual traits

Chuang et al. [29] found that adolescents with high impulsivity traits had a significant positive association with drug addiction. This study also showed that the impulsivity trait alone was an independent risk factor that increased the odds between two to four times for using any drug compared to the non-impulsive group. Another longitudinal study by Guttmannova et al. showed that rebellious traits are positively associated with marijuana drug abuse [27]. The authors argued that measures of rebelliousness are a good proxy for a youth’s propensity to engage in risky behavior. Nevertheless, Wilson et al. [37], in a study involving 112 youths undergoing detoxification treatment for opioid abuse, found that a majority of the affected respondents had difficulty in regulating their emotions. The authors found that those with emotional regulation impairment traits became opioid dependent at an earlier age. Apart from that, a case-control study among outpatient youths found that adolescents involved in cannabis abuse had significant alexithymia traits compared to the control population [28]. Those adolescents scored high in the dimension of Difficulty in Identifying Emotion (DIF), which is one of the key definitions of diagnosing alexithymia. Overall, the adjusted Odds Ratio for DIF in cannabis abuse was 1.11 (95% CI, 1.03–1.20).

#### Significant negative growth exposure

A history of maltreatment in the past was also shown to have a positive association with adolescent drug abuse. A study found that a history of physical abuse in the past is associated with adolescent drug abuse through a Path Analysis, despite evidence being limited to the female gender [25]. However, evidence from another study focusing at foster care concluded that any type of maltreatment might result in a prevalence as high as 85.7% for the lifetime use of cannabis and as high as 31.7% for the prevalence of cannabis use within the last 3-months [30]. The study also found significant latent variables that accounted for drug abuse outcomes, which were chronic physical maltreatment (factor loading of 0.858) and chronic psychological maltreatment (factor loading of 0.825), with an r^2^ of 73.6 and 68.1%, respectively. Another study shed light on those living in child welfare service (CWS) [35]. It was observed through longitudinal measurements that proportions of marijuana usage increased from 9 to 18% after 36 months in CWS. Hence, there is evidence of the possibility of a negative upbringing at such shelters.

#### Personal psychiatric diagnosis

The robust studies conducted in the USA have deduced that adolescents diagnosed with a conduct problem (CP) have a positive association with marijuana abuse (OR = 1.75 [1.56, 1.96], *p* < 0.0001). Furthermore, those with a diagnosis of Major Depressive Disorder (MDD) showed a significant positive association with marijuana abuse.

#### Previous substance and addiction history

Another study found that exposure to e-cigarettes within the past 30 days is related to an increase in the prevalence of marijuana use and prescription drug use by at least four times in the 8th and 10th grades and by at least three times in the 12th grade [34]. An association between other behavioral addictions and the development of drug abuse was also studied [29]. Using a 12-item index to assess potential addictive behaviors [39], significant associations between drug abuse and the groups with two behavioral addictions (OR = 3.19, 95% CI 1.25,9.77) and three behavioral addictions (OR = 3.46, 95% CI 1.25,9.58) were reported.

#### Comorbidity

The paper by Dash et al. (2020) highlight adolescent with a disease who needs routine medical pain treatment have higher risk of opioid misuse [38]. The adolescents who have disorder symptoms may have a risk for opioid misuse despite for the pain intensity.

#### Individual’s attitudes and perceptions

In a study conducted in three Latin America countries (Argentina, Chile, and Uruguay), it was shown that adolescents with low or no perceived risk of taking marijuana had a higher risk of abuse (OR = 8.22 times, 95% CI 7.56, 10.30) [35]. This finding is in line with another study that investigated 2002 adolescents and concluded that perceiving the drug as harmless was an independent risk factor that could prospectively predict future marijuana abuse [27]. Moreover, some youth interviewed perceived that they gained benefits from substance use [38]. The focus group discussion summarized that the youth felt positive personal motivation and could escape from a negative state by taking drugs. Apart from that, adolescents who had high-perceived availability of drugs in their neighborhoods were more likely to increase their usage of marijuana over time (OR = 11.00, 95% CI 9.11, 13.27) [35]. A cheap price of the substance and the availability of drug dealers around schools were factors for youth accessibility [38]. Perceived drug accessibility has also been linked with the authorities’ enforcement programs. The youth perception of a lax community enforcement of laws regarding drug use at all-time points predicted an increase in marijuana use in the subsequent assessment period [27]. Besides perception, a study examining the attitudes towards synthetic drugs based on 8076 probabilistic samples of Macau students found that the odds of the lifetime use of marijuana was almost three times higher among those with a strong attitude towards the use of synthetic drugs [32]. In addition, total screen time among the adolescent increase the likelihood of frequent cannabis use. Those who reported daily cannabis use have a mean of 12.56 h of total screen time, compared to a mean of 6.93 h among those who reported no cannabis use. Adolescent with more time on internet use, messaging, playing video games and watching TV/movies were significantly associated with more frequent cannabis use [44].

### Protective factors

#### Individual traits

Some individual traits have been determined to protect adolescents from developing drug abuse habits. A study by Marin et al. found that youth with an optimistic trait were less likely to become drug dependent [33]. In this study involving 1104 Iranian students, it was concluded that a higher optimism score (measured using the Children Attributional Style Questionnaire, CASQ) was a protective factor against illicit drug use (OR = 0.90, 95% CI: 0.85–0.95). Another study found that high levels of mindfulness, measured using the 25-item Child Acceptance and Mindfulness Measure, CAMM, lead to a slower progression toward injectable drug abuse among youth with opioid addiction (1.67 years, *p* = .041) [37]. In addition, the social phobia trait was found to have a negative association with marijuana use (OR = 0.87, 95% CI 0.77–0.97), as suggested [31].

#### Individual’s attitudes and perceptions

According to El Kazdouh et al., individuals with a strong belief against substance use and those with a strong desire to maintain their health were more likely to be protected from involvement in drug abuse [46].

### DOMAIN: family factors

#### Risk factors

The biological factors underlying drug abuse in adolescents have been reported in several studies. Epigenetic studies are considered important, as they can provide a good outline of the potential pre-natal factors that can be targeted at an earlier stage. Expecting mothers who smoke tobacco and alcohol have an indirect link with adolescent substance abuse in later life [24, 39]. Moreover, the dynamic relationship between parents and their children may have some profound effects on the child’s growth. Luk et al. examined the mediator effects between parenting style and substance abuse and found the maternal psychological control dimension to be a significant variable [26]. The mother’s psychological control was two times higher in influencing her children to be involved in substance abuse compared to the other dimension. Conversely, an indirect risk factor towards youth drug abuse was elaborated in a study in which low parental educational level predicted a greater risk of future drug abuse by reducing the youth’s perception of harm [27, 43]. Negligence from a parental perspective could also contribute to this problem. According to El Kazdouh et al. [46], a lack of parental supervision, uncontrolled pocket money spending among children, and the presence of substance-using family members were the most common negligence factors.

#### Protective factors

While the maternal factors above were shown to be risk factors, the opposite effect was seen when the paternal figure equipped himself with sufficient knowledge. A study found that fathers with good information and awareness were more likely to protect their adolescent children from drug abuse [26]. El Kazdouh et al. noted that support and advice could be some of the protective factors in this area [46].

### DOMAIN: community factors

#### Risk factor

A study in 2017 showed a positive association between adolescent drug abuse and peers who abuse drugs [32, 39]. It was estimated that the odds of becoming a lifetime marijuana user was significantly increased by a factor of 2.5 (*p* < 0.001) among peer groups who were taking synthetic drugs. This factor served as peer pressure for youth, who subconsciously had desire to be like the others [38]. The impact of availability and engagement in structured and unstructured activities also play a role in marijuana use. The findings from Spillane (2000) found that the availability of unstructured activities was associated with increased likelihood of marijuana use [42].

#### Protective factor

Strong religious beliefs integrated into society serve as a crucial protective factor that can prevent adolescents from engaging in drug abuse [38, 45]. In addition, the school connectedness and adult support also play a major contribution in the drug use [40].

## Discussion

The goal of this review was to identify and classify the risks and protective factors that lead adolescents to drug abuse across the three important domains of the individual, family, and community. No findings conflicted with each other, as each of them had their own arguments and justifications. The findings from our review showed that individual factors were the most commonly highlighted. These factors include individual traits, significant negative growth exposure, personal psychiatric diagnosis, previous substance and addiction history, and an individual’s attitude and perception as risk factors.

Within the individual factor domain, nine articles were found to contribute to the subdomain of personal/ individual traits [27–29, 37–40, 43, 44]. Despite the heterogeneity of the study designs and the substances under investigation, all of the papers found statistically significant results for the possible risk factors of adolescent drug abuse. The traits of high impulsivity, rebelliousness, difficulty in regulating emotions, and alexithymia can be considered negative characteristic traits. These adolescents suffer from the inability to self-regulate their emotions, so they tend to externalize their behaviors as a way to avoid or suppress the negative feelings that they are experiencing [41, 47, 48]. On the other hand, engaging in such behaviors could plausibly provide a greater sense of positive emotions and make them feel good [49]. Apart from that, evidence from a neurophysiological point of view also suggests that the compulsive drive toward drug use is complemented by deficits in impulse control and decision making (impulsive trait) [50]. A person’s ability in self-control will seriously impaired with continuous drug use and will lead to the hallmark of addiction [51].

On the other hand, there are articles that reported some individual traits to be protective for adolescents from engaging in drug abuse. Youth with the optimistic trait, a high level of mindfulness, and social phobia were less likely to become drug dependent [31, 33, 37]. All of these articles used different psychometric instruments to classify each individual trait and were mutually exclusive. Therefore, each trait measured the chance of engaging in drug abuse on its own and did not reflect the chance at the end of the spectrum. These findings show that individual traits can be either protective or risk factors for the drugs used among adolescents. Therefore, any adolescent with negative personality traits should be monitored closely by providing health education, motivation, counselling, and emotional support since it can be concluded that negative personality traits are correlated with high risk behaviours such as drug abuse [52].

Our study also found that a history of maltreatment has a positive association with adolescent drug abuse. Those adolescents with episodes of maltreatment were considered to have negative growth exposure, as their childhoods were negatively affected by traumatic events. Some significant associations were found between maltreatment and adolescent drug abuse, although the former factor was limited to the female gender [25, 30, 36]. One possible reason for the contrasting results between genders is the different sample populations, which only covered child welfare centers [36] and foster care [30]. Regardless of the place, maltreatment can happen anywhere depending on the presence of the perpetrators. To date, evidence that concretely links maltreatment and substance abuse remains limited. However, a plausible explanation for this link could be the indirect effects of posttraumatic stress (i.e., a history of maltreatment) leading to substance use [53, 54]. These findings highlight the importance of continuous monitoring and follow-ups with adolescents who have a history of maltreatment and who have ever attended a welfare center.

Addiction sometimes leads to another addiction, as described by the findings of several studies [29, 34]. An initial study focused on the effects of e-cigarettes in the development of other substance abuse disorders, particularly those related to marijuana, alcohol, and commonly prescribed medications [34]. The authors found that the use of e-cigarettes can lead to more severe substance addiction [55], possibly through normalization of the behavior. On the other hand, Chuang et al.’s extensive study in 2017 analyzed the combined effects of either multiple addictions alone or a combination of multiple addictions together with the impulsivity trait [29]. The outcomes reported were intriguing and provide the opportunity for targeted intervention. The synergistic effects of impulsiveness and three other substance addictions (marijuana, tobacco, and alcohol) substantially increased the likelihood for drug abuse from 3.46 (95%CI 1.25, 9.58) to 10.13 (95% CI 3.95, 25.95). Therefore, proper rehabilitation is an important strategy to ensure that one addiction will not lead to another addiction.

The likelihood for drug abuse increases as the population perceives little or no harmful risks associated with the drugs. On the opposite side of the coin, a greater perceived risk remains a protective factor for marijuana abuse [56]. However, another study noted that a stronger determinant for adolescent drug abuse was the perceived availability of the drug [35, 57]. Looking at the bigger picture, both perceptions corroborate each other and may inform drug use. Another study, on the other hand, reported that there was a decreasing trend of perceived drug risk in conjunction with the increasing usage of drugs [58]. As more people do drugs, youth may inevitably perceive those drugs as an acceptable norm without any harmful consequences [59].

In addition, the total spent for screen time also contribute to drug abuse among adolescent [43]. This scenario has been proven by many researchers on the effect of screen time on the mental health [60] that leads to the substance use among the adolescent due to the ubiquity of pro-substance use content on the internet. Adolescent with comorbidity who needs medical pain management by opioids also tend to misuse in future. A qualitative exploration on the perspectives among general practitioners concerning the risk of opioid misuse in people with pain, showed pain management by opioids is a default treatment and misuse is not a main problem for the them [61]. A careful decision on the use of opioids as a pain management should be consider among the adolescents and their understanding is needed.

Within the family factor domain, family structures were found to have both positive and negative associations with drug abuse among adolescents. As described in one study, paternal knowledge was consistently found to be a protective factor against substance abuse [26]. With sufficient knowledge, the father can serve as the guardian of his family to monitor and protect his children from negative influences [62]. The work by Luk et al. also reported a positive association of maternal psychological association towards drug abuse (IRR 2.41, *p* < 0.05) [26]. The authors also observed the same effect of paternal psychological control, although it was statistically insignificant. This construct relates to parenting style, and the authors argued that parenting style might have a profound effect on the outcomes under study. While an earlier literature review [63] also reported such a relationship, a recent study showed a lesser impact [64] with regards to neglectful parenting styles leading to poorer substance abuse outcomes. Nevertheless, it was highlighted in another study that the adolescents’ perception of a neglectful parenting style increased their odds (OR 2.14, *p* = 0.012) of developing alcohol abuse, not the parenting style itself [65]. Altogether, families play vital roles in adolescents’ risk for engaging in substance abuse [66]. Therefore, any intervention to impede the initiation of substance use or curb existing substance use among adolescents needs to include parents—especially improving parent–child communication and ensuring that parents monitor their children’s activities.

Finally, the community also contributes to drug abuse among adolescents. As shown by Li et al. [32] and El Kazdouh et al. [46], peers exert a certain influence on other teenagers by making them subconsciously want to fit into the group. Peer selection and peer socialization processes might explain why peer pressure serves as a risk factor for drug-abuse among adolescents [67]. Another study reported that strong religious beliefs integrated into society play a crucial role in preventing adolescents from engaging in drug abuse [46]. Most religions devalue any actions that can cause harmful health effects, such as substance abuse [68]. Hence, spiritual beliefs may help protect adolescents. This theme has been well established in many studies [60, 69–72] and, therefore, could be implemented by religious societies as part of interventions to curb the issue of adolescent drug abuse. The connection with school and structured activity did reduce the risk as a study in USA found exposure to media anti-drug messages had an indirect negative effect on substances abuse through school-related activity and social activity [73]. The school activity should highlight on the importance of developmental perspective when designing and offering school-based prevention programs [75].

### Limitations

We adopted a review approach that synthesized existing evidence on the risk and protective factors of adolescents engaging in drug abuse. Although this systematic review builds on the conclusion of a rigorous review of studies in different settings, there are some potential limitations to this work. We may have missed some other important factors, as we only included English articles, and article extraction was only done from the three search engines mentioned. Nonetheless, this review focused on worldwide drug abuse studies, rather than the broader context of substance abuse including alcohol and cigarettes, thereby making this paper more focused.

## Conclusions

This review has addressed some recent knowledge related to the individual, familial, and community risk and preventive factors for adolescent drug use. We suggest that more attention should be given to individual factors since most findings were discussed in relation to such factors. With the increasing trend of drug abuse, it will be critical to focus research specifically on this area. Localized studies, especially those related to demographic factors, may be more effective in generating results that are specific to particular areas and thus may be more useful in generating and assessing local control and prevention efforts. Interventions using different theory-based psychotherapies and a recognition of the unique developmental milestones specific to adolescents are among examples that can be used. Relevant holistic approaches should be strengthened not only by relevant government agencies but also by the private sector and non-governmental organizations by promoting protective factors while reducing risk factors in programs involving adolescents from primary school up to adulthood to prevent and control drug abuse. Finally, legal legislation and enforcement against drug abuse should be engaged with regularly as part of our commitment to combat this public health burden.

## Data Availability

All data generated or analysed during this study are included in this published article.
